# American Medical Association

**Published:** 1892-07

**Authors:** 


### Editorial.

### THE AMERICAN MEDICAL ASSOCIATION.

The forty-third annual meeting of the American Medical Association was held at Detroit, Mich., on June 7, 8, 9, and 10. The veteran founder, Dr. N. S. Davis, of Chicago, was present, among others. He is now seventy-five years old but a very active man still to be the father of the American Medical Association. There were four Associations of medical men in Detroit, within less than two weeks, and Dr. Davis was conspicuous for his influence in all of them. The meeting that was first held was that of the American Academy of Medicine, which recommended a classical collegiate education, preparatory to the study of medicine. Immediately after the American Medical Association meeting, was that of the medical colleges. All this seems to point to more interest in the education of medical students.

Dr. Davis, as chairman of the judicial committee, brought in a report that Dr. W. W. Potter, who is late president of the Medical Society of the State of New York, could not be recognized as a member and officer of the American Medical Association. The point was made by Dr. Davis and sustained by the Association, that the decision of the judicial committee was final. A motion was adopted, however, to revise the code of ethics. It is possible that this may result in a change of the time-honored position of the American Medical Association in regard to consultations with homœopathic physicians, but it is not probable. At any rate, the present meeting of the Association has taken strong ground against the new code men, and it remains to be seen whether the next meeting will make any radical changes in the Association's policy. There were several prominent New York physicians who had been invited by officers of the Association to read papers. They were anxious to know upon what footing they stood. This was a stage of momentous importance to the Association, and the

President, Dr. H. O. Marcy, and others, are to be congratulated upon the tact displayed in dealing with this question.

Another somewhat sensational episode in the recent meeting, was a hearty denunciation of the Keely cure for inebriety.

The sections were largely attended, and all reports point to renewed interest in the work of the sections. There is consequently no danger that the Association will for the present be supplanted by the special societies that are springing up throughout the United States. There is a work of united effort to be done by the Association that cannot be done by any other society, while there is also a sphere for all.

The next meeting of the Association will be at Milwaukee, Wisconsin. This will be near enough to Chicago to enable the members of the Association to attend the World's Fair at Chicago, and to profit by the reduced rates, and yet during the meeting, the members may be sufficiently isolated for efficient work of a scientific nature.

The officers that will have charge of the Association at Milwaukee, are as follows: Dr. Hunter McGuire, President; Drs. H. O. Walker, H. Brown, H. Janes, Jesse Hawes, Vice-Presidents; Dr. R. J. Dunglison, Treasurer; Dr. W. B. Atkinson, Secretary; Dr. Montgomery, Assistant-Secretary; Dr. George W. Webster, Librarian.

Among the men from the South in attendance upon the Association at Detroit were Drs. J. McFadden Gaston, Floyd W. McRae and W. E. B. Davis, of Georgia; Hunter McGuire, Landon B. Edwards, Hugh M. Taylor, of Virginia; A. N. Talley, cf South Carolina; W. H. Wathen, of Kentucky; W. T. Briggs, of Tennessee, and Jerome Cochrane, of Alabama.

Dr. J. McF. Gaston is to be congratulated on the good attendance and high standard of the Surgical Section over which he presided. Forty papers were read by such men as Gaston, Marcy, Senn, Wyeth, Ransohoff, etc. He is on the National Pan-American Medical Committee, while Dr. F. W. McRae is one of the auxiliary committeemen in Georgia. Dr. W. E. B. Davis, of Rome, Ga., was one of the vice-presidents of the late meeting, and read a paper in the Section of Surgery and Anatomy on "Peritonitis from Gall-Stones." He is also on the judicial committee for the next year.

The Medical Editors' Association had a meeting in Detroit, and also a banquet, both of which were greatly enjoyed.

The *International Journal of Surgery* has an admirable report of the Surgical Section, and of the papers on Gall Bladder Surgery by Drs. Gaston, Myers, and Davis. It is signed by A. D. Among other things we find the following paragraph:

"Discussion on these three papers was then opened by Dr. A. Vander Veer, of Albany, N. Y., who congratulated Dr. Gaston on the importance and value of his work in the surgery of the gall bladder, and on the proud position he occupied both here and abroad. In the treatment of gall-stones in general he was in favor of first trying medical treatment, such as olive oil, etc., but when that failed, then surgical treatment was necessary."

We also quote from the editorial in the same journal:

"The meetings of the Section on Surgery were well attended, and the proceedings were ably conducted under the efficient management of Dr. Gaston, the Chairman. A large number of valuable papers were presented, but time did not permit of sufficient discussion to bring out the salient points of the papers read. This was a source of egret to many of the members who were anxious to discuss them. As Dr. Gaston justly remarked, a full discussion is frequently of greater importance than the paper which has brought it out.

"The appointment of Dr. Hunter McGuire, of Richmond, as President of the Association for 1893, was received with general satisfaction, and is a graceful compliment to one of the foremost surgeons of this country."

